# Epidermal stem cells in wound healing and their clinical applications

**DOI:** 10.1186/s13287-019-1312-z

**Published:** 2019-07-29

**Authors:** Ronghua Yang, Fengxia Liu, Jingru Wang, Xiaodong Chen, Julin Xie, Kun Xiong

**Affiliations:** 1 Department of Burn Surgery, The First People’s Hospital of Foshan, Foshan, 528000 China; 2 Department of Human Anatomy, School of Basic Medical Science, Xinjiang Medical University, Urumqi, 830001 China; 3 Department of Burn Surgery, First Affiliated Hospital of Sun Yat-Sen University, Guangzhou, 512100 China; 4 Department of Anatomy and Neurobiology, School of Basic Medical Science, Morphological Sciences Building, Central South University, 172 Tongzi Po Road, Changsha, 410013 Hunan China

**Keywords:** Epidermal stem cells, Wound healing, Signaling pathway, Epithelial regeneration

## Abstract

The skin has important barrier, sensory, and immune functions, contributing to the health and integrity of the organism. Extensive skin injuries that threaten the entire organism require immediate and effective treatment. Wound healing is a natural response, but in severe conditions, such as burns and diabetes, this process is insufficient to achieve effective treatment. Epidermal stem cells (EPSCs) are a multipotent cell type and are committed to the formation and differentiation of the functional epidermis. As the contributions of EPSCs in wound healing and tissue regeneration have been increasingly attracting the attention of researchers, a rising number of therapies based on EPSCs are currently under development. In this paper, we review the characteristics of EPSCs and the mechanisms underlying their functions during wound healing. Applications of EPSCs are also discussed to determine the potential and feasibility of using EPSCs clinically in wound healing.

## Introduction

As the largest organ and first barrier in the body, the skin has multiple important functions, such as preventing pathogens and dehydration, regulating body temperature, and supplying sensation. The skin is also an active immune organ, hosting cellular elements of the innate and adaptive immune systems [1]. Skin wound healing is a highly organized process that leads to the restoration of tissue integrity and functions. Aberrations of wound healing consume substantial resources and often require long-term medical management [2]. Serious and widespread skin damage, such as burn injury, threatens the entire organism and impairs the capacity for skin regeneration. Moreover, with the increased prevalence of such diseases as diabetes, vascular disease, and obesity, chronic wounds are becoming a major global issue with limited treatment strategies, unsatisfactory therapeutic effects, and significant medical costs [3].

The skin exhibits tremendous regenerative potential because different types of stem cells (SCs) are located in the skin and its appendages; these SCs maintain skin homeostasis and regulate skin damage under physiological conditions. Among these SCs, epidermal stem cells (EPSCs) are of particular interest because they are numerous and accessible. In addition, EPSCs are easy to obtain without potential ethical and political issues compared to embryonic stem cells, which are similar to adipose-derived stem cells, a cell type that has been widely used in regenerative medicine and clinical studies [4]. EPSCs have been studied for possible regenerative approaches since the 1970s to overcome the limitations of conventional therapeutic strategies. Several approaches based on EPSCs have been demonstrated that can promote wound healing or replace irreversibly lost skin, and some of them have advanced into clinical applications [5].

In this review, we aim primarily to outline the populations of EPSCs and their characteristics. In the following sections, we present the important roles of EPSCs during wound healing and discuss the associated mechanisms that regulate their activities. We finally focus on the relevance of EPSCs in the context of wound healing and epithelial damage in other organs and discuss the potential clinical applications of these cells.

## Populations of EPSCs

The epidermis is composed of the interfollicular epidermis (IFE) to the infundibulum and contains appendages including hair follicles (HFs), sebaceous glands (SGs), and sweat glands [6]. Each compartment has its own specialized SCs capable of maintaining tissue growth independently [7, 8]. The specific microenvironment in which EPSCs reside is named a niche, which is composed of various cell types and is important for modulating SC activity by cell contact, extracellular matrix (ECM) components, and growth factors [9, 10]. Three distinct EPSC niches, including the basal layer of the epidermis, the bulge region of the HF (distinct region in mice but not in humans), and the base of the SG shaft, were identified in the skin [10–12].

**Fig. 1:**
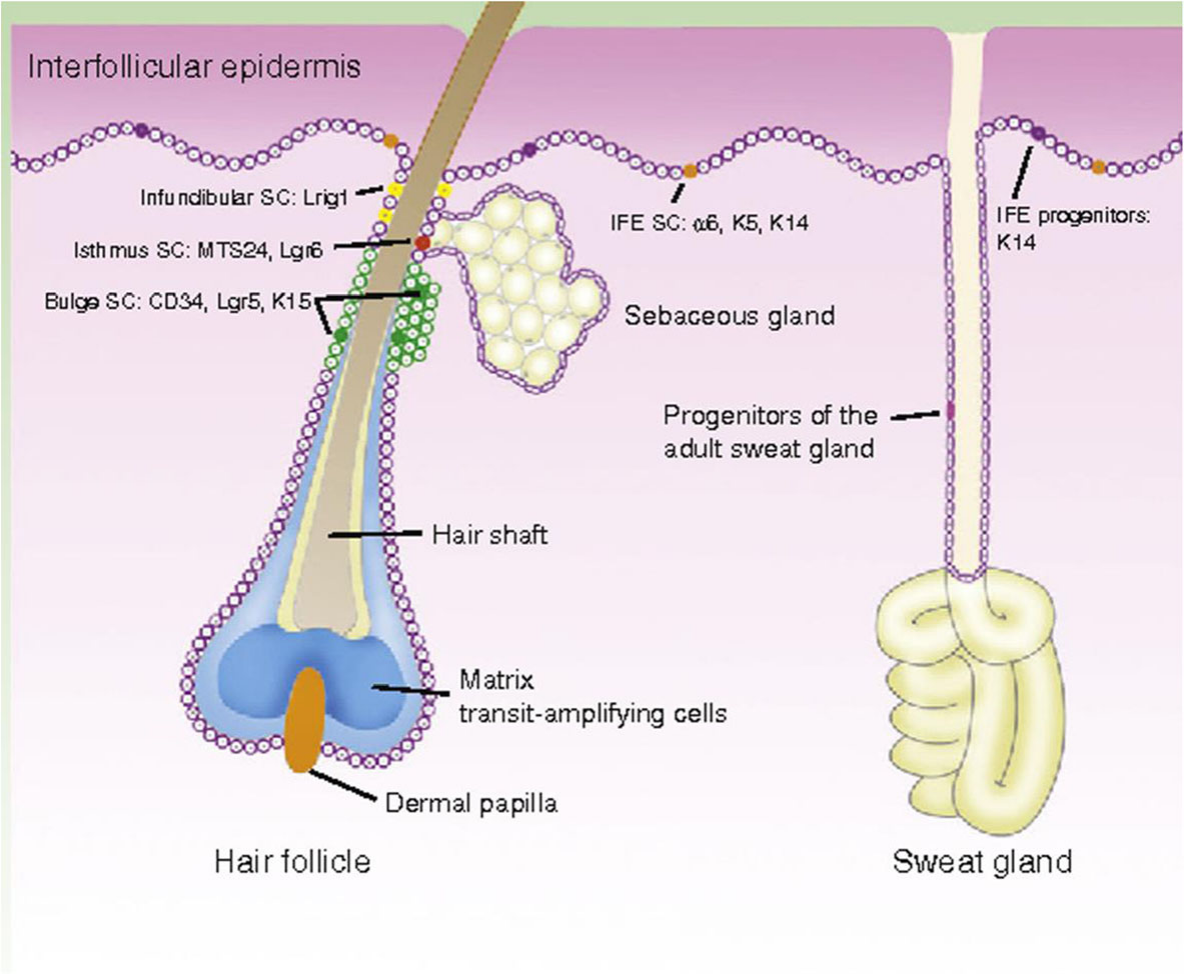
Illustration of the different populations of EPCs and their specific markers

The EPSCs that are located in different niches have their own markers and functions (Fig. 1). IFESCs are located in the basal layer of the IFE and give rise to suprabasal, differentiated cells. IFESCs express high levels of β1 and α6 integrins, Leu-rich repeats and immunoglobulin-like domains (LRIG)1, and melanoma-associated chondroitin sulfate proteoglycan [13–15]. These cells can also be traced using K14-CreER or Inv-CreER mouse strains induced at low dose [16, 17]. IFESCs not only replenish the basal layer but also give rise to nonproliferative, transcriptionally active spinous and granular layers and, finally, the outer layers of terminally differentiated stratum corneum [13, 18]. HFSCs reside in the permanent noncyclic follicle portion named the bulge [19] and possess specific bulge markers, such as CD34 [19], keratin (KRT)15/19 [20, 21], leucine-rich-repeat-containing G protein-coupled receptor (LGR)5 [22], SRY-box (SOX)9 [23], and transcription factor (TCF)3 [24]. HFSCs, the first identified EPSCs based on their slow cycling properties [25, 26], are reported to have higher clonogenicity and give rise to IFE, HF, and SG lineages upon transplantation [19, 27]. However, in marked contrast to transplantation experiments, lineage tracing studies have shown that HFSCs only contribute to HF regeneration and do not sustain the IFE, SG, or infundibulum [23, 24]. In addition to IFESCs and HFSCs, some other EPSC populations are located in different skin appendages. For example, infundibulum SCs expressing LRIG1 are located in the upper part of the isthmus [28, 29]. SG duct SCs are located at the entrance of the gland and express GATA-binding protein (GATA)6 [29]. Basal cells, including isthmus and SG SCs, are located at the junction between the HFs and the gland; express LRIG1, LGR6, and PR/SET domain (PRDM)1; and give rise to the entire SG and isthmus [30]. All of the pools of EPSCs contribute to epidermal homeostasis and wound healing.

## Characteristics of EPSCs

### EPSC plasticity

Individual EPSC populations exhibit considerable differentiation potential, which is defined as plasticity [31]. When IFESCs and HFSCs are recruited to the IFE after injury, they progressively lose their initial identities and differentiate into the IFE fate [32]. Studies have indicated that long-lived IFESCs are recruited to the wound area and promote re-epithelialization after injury, while short-lived involucrin+ IFESCs also migrate to wound sites. Most involucrin+-derived progeny terminally differentiate within a month [13], suggesting that lineage reversion is not sustained for an extended period. The transient nature of lineage reversion observed in IFE contrasts with findings made in the esophagus, where progenitors change their mode of proliferation in repairing incisional wounds [33]. Apart from IFESCs, HFSCs also exhibit plasticity in response to damage. SCs in the bulge and infundibulum migrate upward, proliferate, and participate in the epidermal repair process [34]. The migrating HFSCs progressively lose their initial identities and adopt an IFE differentiation program. However, these cells do not persist for extended periods within IFE [34].

Differentiated suprabasal epidermal cells can revert back to SCs upon wounding [35, 36]. A population of GATA6-positive cells that reside in the isthmus give rise to the sebaceous duct in the steady-state condition. During wound healing, these cells are mobilized, migrate to the injured IFE, and revert from a differentiated fate to a basal SC fate and acquire SC properties [29]. The phenomenon of dedifferentiation has also been observed in HFs. After depilation or targeted ablation to induce the loss of bulge HFSCs, differentiated hair germ cells, infundibulum, or SG cells can repopulate the SC niche and establish functional HFSCs [37, 38].

### EPSC self-renewal, dynamics, proliferation, and migration

SCs can produce differentiated cells, but they also propagate to maintain a constant pool of SCs by dividing symmetrically or asymmetrically [39]. During embryo development, most basal cell divisions are symmetric (an SC gives rise to two identical daughter cells, exhibiting either a differentiated or a somewhat differentiated phenotype) and parallel to the axis of the basal membrane, enabling the growth of the embryo surface and ensuring that the epithelium remains as a single layer. In contrast, during stratification of the epithelium, which occurs in homeostasis in adulthood, ~ 70% of divisions are asymmetric (a daughter cell, on losing contact with integrins and growth factor secreted by the basal membrane, undergoes differentiation, and the second daughter cell, on remaining in contact with the basal membrane, maintains the characteristics of the SC) [40], thereby allowing development of suprabasal cells and establishment of the epidermis [17, 41]. However, during wound healing, cell numbers need to increase to compensate for the injured and lost cells. Excess renewal over differentiation can be achieved by enhancing symmetric renewal or decreasing the proportion of cells that undergo differentiation [42].

Increased keratinocyte proliferation can be observed during wound healing [43]. However, cell proliferation can only be detected in a proliferative zone (0.5–1.5 mm away from the edge), not at the wound edge, where cells located in basal and suprabasal layers migrate as a cellular sheet [44, 45]. Cells that are closer to the leading edge have the greatest migration speed, and the speed decreases thereafter. Both proliferation and migration co-occur at a distance of 0.5 mm from the wound edge, where basal cells are elongated toward the wound and orient their division in this direction [45]. At present, it is still unknown whether cell proliferation is essential for migration. Studies using mouse tail skin have suggested that inhibition of cell proliferation prevents wound closure and cell compression at the leading edge [44]. However, cells became more elongated upon inhibition of proliferation, suggesting an important compensatory phenomenon [45]. Therefore, cell migration at the leading edge comes first, and the displacement of the cell gives rise to the oriented division of the cells following behind. Enhanced proliferation can generate a surplus of migrating cells that later push the leading edge toward the wound center [16]. Additionally, keratinocytes that acquire migratory phenotypes inhibit characteristics of epithelial-to-mesenchymal transition (EMT), such as decreased expression of cell adhesion molecules, enhanced motility, and increased expression of EMT markers [46, 47], which are essential to wound healing.

## Role of EPSCs in cutaneous wound healing

### Events in normal cutaneous wound healing

Skin wound healing is imperative to restore skin defects and to regain lost integrity, tensile strength, and barrier function [48]. Wound healing is a multifaceted and highly regulated process that endows various cell types with essential functions; this process is commonly divided into four successive but overlapping phases [49, 50].

#### Hemostasis

Upon injury, platelets are exposed to the ECM proteins, leading to instant coagulation and fibrin clot formation, which functions as a provisional wound matrix. In addition, platelets also activate the following inflammation phase through secreting growth factors [51]. In addition, the proteins that fill the blood clot, such as thrombospondin, vitronectin, and fibronectin, facilitate the migration of wound-healing-related cells, including keratinocytes, blood cells, and endothelial cells [52].

#### Inflammation

Neutrophils, the first cells that arrive at the wound site, clear debris and bacteria, secrete cytokines [e.g., interleukin (IL)-1α, IL-1β, and tumor necrosis factor (TNF)-α)] to attract and activate other cells, and amplify the inflammatory cascade [53–55]. Macrophages then migrate to the wound, clean up pathogens, and promote keratinocyte migration and ECM synthesis by secreting cytokines and growth factors, such as transforming growth factor (TGF)-α, TGF-β, fibroblast growth factors (FGFs), and platelet-derived growth factor (PDGF) [49].

#### Proliferation

The subsequent proliferative phase is characterized by granulation tissue formation, which replaces the original coagulation and fibrin clot and provides a wound bed for re-epithelialization. In this stage, bidirectional interactions between keratinocytes and fibroblasts are essential, and a paracrine signaling loop exists between them, which promotes keratinocyte proliferation and the fibroblast secretion of cytokines and growth factors important in wound healing [50, 56].

#### Tissue remodeling

In the last phase of wound healing, fibroblasts differentiate into myofibroblasts stimulated by TGF-β1 and other growth factors, promoting wound contraction and resulting in a reduction in the size of the wound area [57]. The granulation tissue degrades, which makes the mature wound tissue avascular and acellular, which is also known as scar formation [52].

### Function of EPSCs in cutaneous wound healing

Robust activation of EPSCs and efficient recruitment of their progeny toward an epidermal lineage are critical for re-epithelialization [58], a stage that is also called the re-establishment of an intact keratinocyte layer during wound healing [52].

Under homeostatic conditions, the major epidermal compartments are rejuvenated by differentiation of their own SCs. IFE and SGs undergo constant self-renewal, whereas HFs undergo cycles of phases, including resting, growth, and involution. Each discrete SC niche behaves unipotently, replenishing its own compartment [14, 19, 59]. However, during wound healing, SCs have acquired the ability to repair neighboring compartments, and these compartments are capable of repopulating one another [60]. The manner in which SCs respond to injury varies drastically, depending not only on the specific niches where these reside but also on how close they are to the wound [61].

During wound healing, EPSCs are activated and recruited from different skin regions when spatial confinement and lineage restriction of resident skin SCs are transiently lost, allowing them to contribute to multiple EPSCs [13]. When HFSCs migrate toward the epidermis, they lose their specific markers and adopt a phenotype similar to that of IFESCs. However, once in the epidermis, these cells are short-lived and disappear soon after the damaged tissue is repaired [13]. Studies have shown that HFSCs temporarily contribute to wound re-epithelialization soon after damage but disappear several weeks later, suggesting that HFSCs serve as a transient bandage that allows other SCs from the IFE and upper isthmus/infundibulum to maintain long-term repair [62]. The role of HFSCs was further defined by other researchers, who indicated a delay in the early stage of re-epithelialization when incisional wounds were created in HF-deficient mice, presumably through recruitment of IFESCs and indicating their capability for tissue regeneration [63]. In addition, glabrous skin, such as the ventral part of the paw, heals properly with slower kinetics than human skin does, suggesting that HFSCs are dispensable for wound healing [64]. These studies suggest that the injury-induced vacant niches activate a broad range of SCs to assume characteristics that differ from their homeostatic roles.

#### Regeneration of SGs and sweat glands

The SGs are an important structure in the epidermis, which is closely associated with the hair follicle, and they constitute what is known as the pilosebaceous unit. Lineage tracing reveals that different follicle stem cell populations located above the bulge, including LGR6+ cells, KRT15+ cells, and junctional zone stem cells, may give rise to the entire SGs structures [65]. This population is also multipotent and can replace HF and IF epidermis [65]. Other studies indicate that cells expressing Blimp1 can give rise to terminally differentiated sebocytes though transient amplifying progenitors [30]. Unlike LGR6/KRT15+ cells, Blimp1+ cells do not contribute progeny toward HF or IF epidermis. As a transcriptional repressor, Blimp1 inhibits the transcription of c-Myc, limiting the input of proliferative progenitors toward the gland from the multipotent stem cell populations of the isthmus and bulge [66, 67]. The negative control role of Blimp1 on gland growth was confirmed upon epithelial Blimp1 suppression, which resulted in SG hypertrophy and an oily hair coat phenotype [30]. Unlike the hair follicle and SGs, the sweat glands are relatively quiescent, and few studies have investigated their regenerative potential until recently [21]. Sweat glands are a secretory type of ectodermal appendage and consist of the secretory glandular portion and the duct connected to the skin surface. Transgenic lineage tracing studies suggest the existence of basal myoepithelial and suprabasal luminal progenitor cell populations, which comprise the glandular portion of the sweat gland and can regenerate their own lineage [21]. Glandular cells do not contribute progeny toward the duct which, in turn, is maintained by its own basal unipotent progenitors. The ductal cells but not the glandular cells become activated upon skin injury and contribute to restoring ductal openings [21]. Ductal cells not only regenerate the duct itself but also regenerate glabrous epidermis surrounding the sweat gland opening. A study using 3D reconstruction technology showed that ductal progenitors contribute to the regeneration of human IFE, at least matching that from hair follicles [68]. In this respect, ductal progenitors share characteristics with hair follicle isthmus stem cells, which can generate permanent epidermal progeny upon skin injury [69].

## Mechanism to regulate EPSC function during cutaneous wound healing

### Signaling pathway

**Fig. 2:**
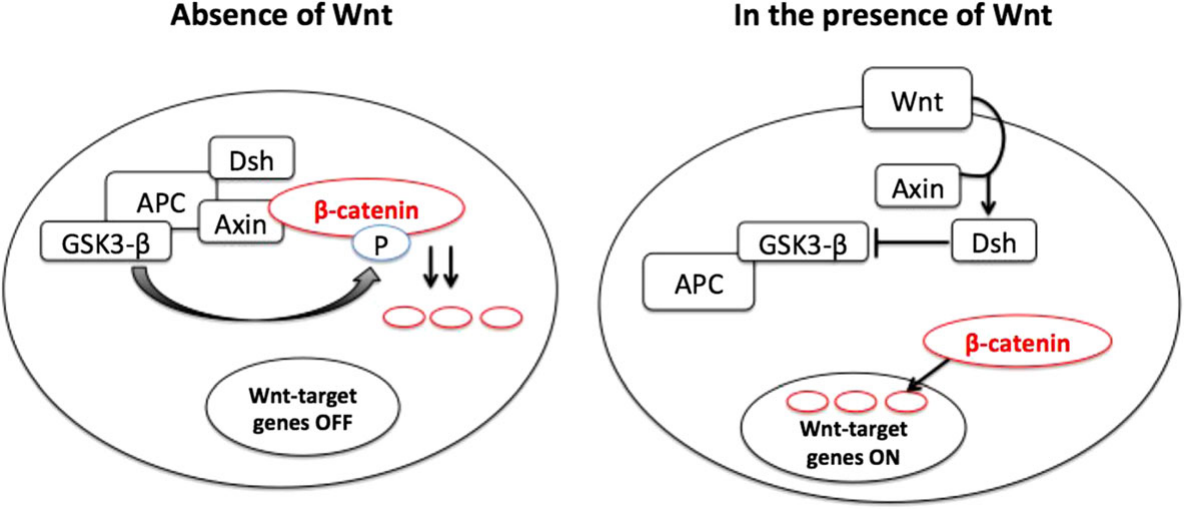
Schematic of the canonical Wnt signaling pathway. In the absence of Wnt signaling (left), β-catenin binds to axin, APC, and GSK3-β and becomes phosphorylated and targeted for degradation. β-Catenin also exists in a cadherin-bound form and regulates cell–cell adhesion. In the presence of Wnt signaling (right), β-catenin is uncoupled from the degradation complex and translocates to the nucleus, where it binds transcription factors, thereby activating target genes

The behavior of EPSCs is directed at multiple levels in response to activating and inhibiting signals. Signaling pathways, such as the Wnt/β-catenin, Sonic hedgehog, Notch, and TGF-β/BMP pathways, as well as Nanog, MAPK, and c-Myc, are involved in the regulation of cell function [70]. In the mediation of self-renewal and differentiation of EPSCs, Wnt signaling is either β-catenin dependent (β-catenin interacts with other transcription pathways, including Sox family members and forkhead box O) or β-catenin independent [71] (Fig. 2). The canonical Wnt pathway functions by inhibiting glycogen synthase kinase 3β, leading to accumulation of unphosphorylated catenin, which then acts as a nuclear cofactor for the lymphoid enhancer binding factor 1/TCF family of DNA-binding proteins [72]. Wnt can promote the accumulation of key microtubule-binding proteins that stabilize microtubules at the wound edge in an active state to promote wound repair [73]. The small GTPase and actin regulator Rac family small GTPase 1 also participate in the solidification of the cytoskeleton in cell migration and wound healing [45].

**Fig. 3:**
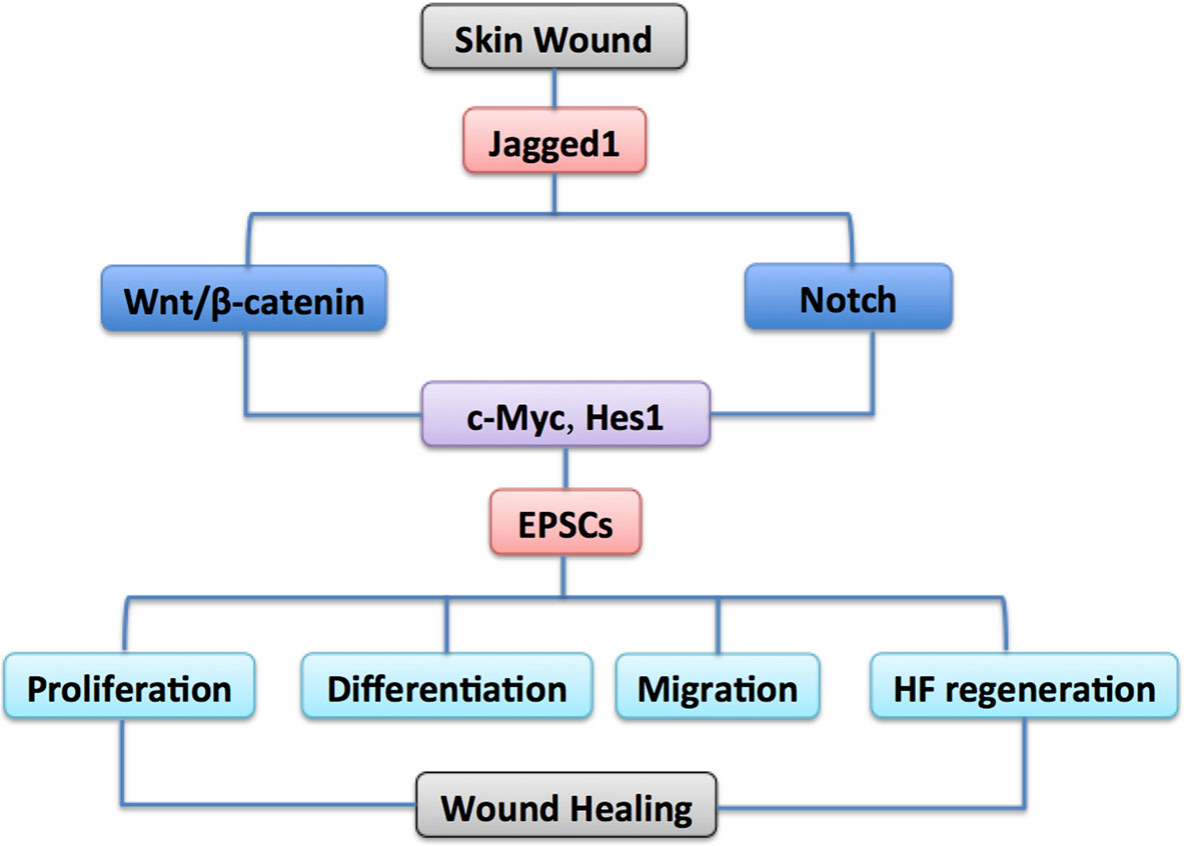
Schematic of crosstalk between the Wnt and Notch pathways and their function in regulating EPSCs during wound healing. Wnt and Notch signaling can affect the proliferation, differentiation, and migration of EPSCs, as well as HF regeneration by targeting c-Myc and Hes1, which ultimately lead to enhanced or delayed wound healing. The interaction between the Wnt and Notch pathways plays a vital role in wound healing, and jagged1 is the key mediator in this crosstalk

Our research group has also studied the signaling pathways that regulate the function of EPSCs during wound healing. By using gain-of-function technology, we found that activation or inhibition of Wnt and Notch signaling can affect proliferation of EPSCs, differentiation and migration of keratinocytes, and HF regeneration by targeting MYC proto-oncogene, bHLH transcription factor (c-Myc), and Hes1, which ultimately lead to enhanced or delayed wound healing. We also found that the interaction between the Wnt and Notch pathways might play a vital role in the regulation of wound healing, and jagged1 may be the key mediator in this crosstalk (Fig. 3) [74].

In addition to multiple signaling pathways that affect EPSCs, factors that trigger coordinated SC fate switching toward epidermal lineage at the onset of wound healing have also been discovered. LIM homeobox (LHX2), Lim-homeodomain transcription factor, which is expressed in bulge SCs and secondary hair germ progenitors, plays a key role in directing follicular SCs toward re-epithelialization [75]. Persistent LHX2 signaling activation in EPSCs is necessary for epidermal fate switching, a process that involves upregulation of SOX9 and TCF4, which function as positive mediators of wound re-epithelialization [76]. Studies from our research group have demonstrated that caveolin-1 plays a critical role in the regulation of EPSC proliferation. Overexpression of caveolin-1 in EPSCs promotes re-epithelialization in wounds, enhances cellularity, and increases vasculature and wound scores, indicating that modification of such genes as caveolin-1 may be an effective approach for promoting EPSC-based therapy in wound healing [77].

### Role of EPSC biomarkers

#### Integrins

Integrins are transmembrane adhesion proteins that are involved in ECM assembly, apoptosis, TGF-β signaling, and cytoskeleton organization during wound healing [78]. β1-Integrins are necessary for re-epithelialization because β1-integrin null mice show decreased migration and excessive hyperproliferation of EPSCs [78]. In addition, β3-integrins were also found to increase cell differentiation by knockdown of miR-378a [79]. Inappropriate ECM composition and mechanics are involved in the pathology of non-healing wounds. Integrins α6β4 and α5β1 cluster at the leading edge of the epidermal cells, where they polarize cell shape and cytoskeletal movements that are needed for cell migration and fibronectin assembly [44].

#### Cadherins and catenins

Cadherins are Ca^2+^-dependent transmembrane glycoproteins that are connected to cytoplasmic β-catenin coupled to α-catenin molecules, and the cadherin–catenin–actin complexes ensure mechanical adhesion of epithelial cells [80]. During wound repair, E-cadherin expression is reduced and alters cell adhesion, promoting cell migration. However, overexpression of E-cadherin makes cells become more tightly packed, thereby preventing cell migration and consequently re-epithelialization [81]. While cadherins are important in the formation of extracellular connections, catenin proteins form a bridge between transmembrane cadherin and intracellular actin filaments. β-Catenin is a key mediator of the Wnt signaling pathway, which can activate HFSC activity and promote regeneration of HF during re-epithelialization [55]. Conversely, it was found that β-catenin and c-myc activation inhibits the migration and differentiation of epidermal cells, leading to the development of chronic wounds [82].

#### Keratins and p63

As an intermediate filament, keratin is important in the durability of the cytoskeleton in epidermal cells. The expression of keratin isotypes 6, 16, and 17 is increased, and the expression of keratin isotypes 1 and 10 is reduced, around epidermal wound sites; these isotypes are essential for wound healing [83]. p63 can regulate the differentiation and proliferation of EPSCs and is involved in wound healing [84]. Phosphorylated p63 levels in the wound area are increased, indicating that EPSCs have differentiated into various progenitors, accompanied by re-epithelialization of the skin tissue [84].

### Epigenetic regulation

#### DNA methylation

During wound healing, DNA methylation occurs specifically in regulatory regions of developmental genes. It has been shown that DNA methyltransferase (DNMT)1 is expressed in the HFs and the basal layer of the epidermis, and its expression rapidly decreases on differentiation [85]. Ablation of DNMT1 from mouse epidermis results in sebaceous hyperplasia, thickened epidermis, and upregulation of some differentiation markers [86]. Animals lacking DNMT1 in the epidermis display premature and progressive alopecia during aging as a result of reduced proliferation and increased apoptosis in the HFSCs [86]. Concordantly, depletion of ubiquitin-like with PHD and ring finger (UHRF)1, a protein that is expressed in undifferentiated basal cells and aids DNMT1 in hemimethylated DNA, also leads to upregulation of differentiation genes and decreased proliferation [72]. The activity of DNMT1/UHRF1 in EPSCs is essential to maintain the balance between preventing excessive differentiation and allowing SC proliferation by repressing genes that block cell-cycle progression [87].

#### Chromatin modification

The differential outcomes of EPSCs during wound healing are regulated by epigenetic mechanisms. Histone deacetylases (HDACs) and histone methyltransferases (HMTs) are important in epidermal and hair follicular development. The absence of specific HMTs, such as enhancer of zeste 1 (*Ezh1*) and enhancer of zeste 2 (*Ezh2*), blocks hair follicular morphogenesis and delays wound healing [88]. Epigenetics influences wound healing in all four phases by regulating the repair machinery at transcriptional and posttranslational levels [89]. The epigenetic events that influence early healing stages are decreased global methylation through reduction of histone H3 lysine 27 (H3K27) trimethylation, downregulation of the polycomb group, and upregulation of histone demethylases [90]. Decreased H3K27 trimethylation enhances the inflammatory process by promoting IL-12 expression, which can be observed in chronic wounds, such as diabetic wounds [91]. During chronic wound healing, increased sirtuin levels and decreased class I HDAC levels enhance the expression of α-tubulin associated with increased H3K9 levels [92]. The epigenetic combinations enhance the proliferation and differentiation of epidermal cells, fostering wound repair that is NO dependent [93]. Alternatively, increases in histone acetyltransferases, such as P300/CBP-associated factor, promote wound healing through processes independent of NO [93].

#### miRNAs

miRNAs are small, noncoding RNAs that regulate gene expression posttranscriptionally, which play key roles in epidermal development and skin SC maintenance [94, 95]. miR-203 plays a significant role in skin morphogenesis and EPSC differentiation and inhibits “stemness” by inhibiting the expression of p63 [96]. Our research group has found that pre-miR-203 treatment increases EPSC differentiation to myofibroblasts, as indicated by decreased K15 expression and increased myofibroblast biomarkers. This phenomenon is reversed by overexpression of the hes family bHLH transcription factor (Hes1) in EPSCs. In addition, skin incision increases the expression of miR-203, and local treatment with miR-203 inhibitor accelerates wound healing and reduces scar formation by increasing Hes1 expression [97]. The importance of miR-125b in the regulation of EPSCs has also been demonstrated [98]. miR-125b expression is increased in the “stem” state but reduced in early skin SC progeny. Overexpression of miR-125b inhibits EPSC proliferation, while knockdown induces proliferation and delays differentiation via fibroblast growth factor receptor 2 regulation. In addition, miR-184 has been shown to induce Notch activation and epidermal differentiation. miR-184 regulates the transition of EPSCs from proliferation to early differentiation, while misexpression or mutation in miR-184 results in impaired skin homeostasis [99].

## Applications of EPSCs

**Fig. 4:**
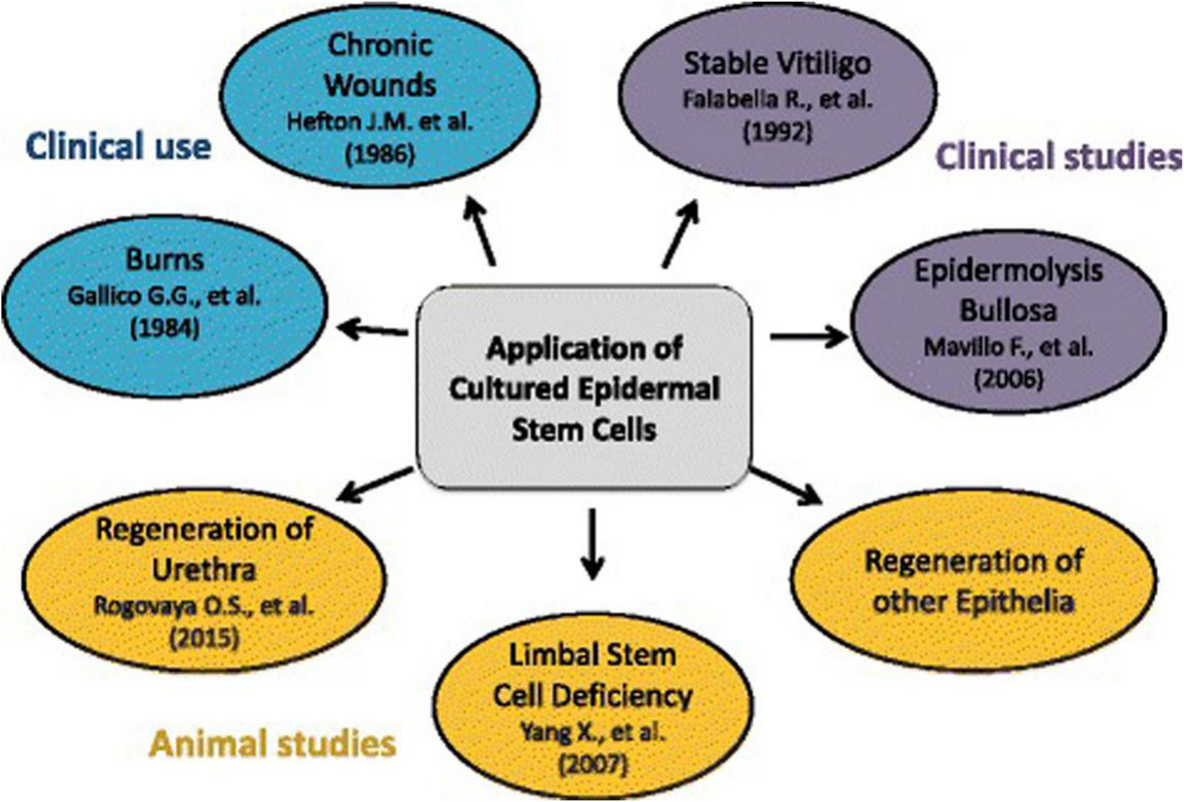
Potential uses of EPCs in the treatment of skin wounds and regeneration of other epithelia in the body

Serious damage that exceeds the regenerative capacity of the skin threatens the entire organism and requires effective treatment methods. Treatment of wounds must be long-lasting, requires specific medical skills, and constitutes a major challenge. EPSCs are considered a convenient target for use in wound management because their advantages include accessibility, simple isolation, and skin regenerative capacity. The cells have been of interest for wound healing since the 1970s. A number of therapeutic strategies have already been developed, and some of them have advanced into the clinical arena (Fig. 4) [100].

### Application of EPSCs in wound management

Cells isolated from skin biopsies are propagated and cultivated on biomaterials, which are called cultured epidermal autografts (CEAs). CEAs are derived from unpurified epidermal cell cultures that are thought to contain EPSCs, which were first used in wound treatment in 1981 and have been available commercially since 1988. Enrichment with EPSCs within CEA has been used to treat serious wounds [101, 102]. Compared with conventional skin autografts, CEAs do not require a second skin injury, which itself can be prone to such complications as pain, infection, delayed healing, and scar formation [103]. Thus, the scalp is an ideal skin source, as it contains abundant HFs, which can lead to better epithelialization. A commercially available autologous product generated from HFSCs, known as EpiDex®, has been applied clinically since 2004, which is ideally suited for chronic wounds exhibiting granulation but not for re-epithelialization. Long-term clinical trials have reported that EpiDex can induce complete wound healing within a 9-month surveillance period [104]. Studies have shown that when EPSCs are cultivated in fibrinogen-derived fibrin glue, wounds that are treated with this system heal with minimal contraction and maintain excellent tissue flexibility. Therefore, this approach is feasible for wounds under intensive or complex mechanical stresses, such as the fingers, toes, and eyelids [105]. In addition, EPSCs cultured in the fibrinogen matrix can form a stable cell layer, which is easy to operate during transplantation and displays adhesion to the wound surface. The combination of EPSCs with allografts is also possible, as the top silicon layer of alloderm can be removed and replaced by a CEA [102]. Another approach is the preparation of suspensions of autologous EPSCs, which can be directly sprayed onto the wounds [103]. These cells are suitable for superficial wounds of small to moderate size, and they can significantly improve wound healing and reduce scar formation. However, this procedure is not ideally suited for large or deep wounds. Additionally, subcutaneous injection of allogeneic EPSCs has also been used by our research group, which can shorten the wound healing period [106].

Aside from the traditional approach of administering living SCs directly to wounds, which has been reported to have many limitations, including low cell survival, high cell attrition rate, difficulties in tissue targeting, and additional tissue damage, new trends have emerged in recent years [107]. Since SCs have strong paracrine capacities and secrete many factors into the medium, such as cytokines, chemokines, growth factors, and other bioactive proteins that are well-known as enhancing factors in wound healing, application of the conditioned (culture) medium (CM) from SCs has been confirmed to facilitate tissue regeneration process [108]. In addition, the extracellular vesicles (EVs), such as exosomes, can be isolated from cultured supernatants of many SC types and exhibit promise as novel therapies against delayed wound healing [109]. Studies have shown that SC-derived exosomes can promote cell migration, angiogenesis, proliferation, and re-epithelialization by activating some signaling pathways, such as STAT3, AKT, ERK, and the Wnt/β-catenin pathway, resulting in the upregulated expression of many growth factors [110]. Furthermore, in vivo studies have also demonstrated that exosomes derived from SCs not only can accelerate wound closure and angiogenesis but are also essential for skin rejuvenation and show potential applications in the field of skin regeneration and cosmetics [111].

### Use of EPSCs in skin wound repair

#### Burn wounds

In extensive burns, less skin is available for split-skin grafts, and skin substitutes are used. Commercial skin substitutes consist of a matrix component used with or without cells. The impermanent nature and simplistic matrix architecture that lacks appropriate mechanical properties are limitations of skin substitutes [112, 113]. Meanwhile, the failure of CEA engraftment is associated with low EPSC content. Thus, transplants of skin substitutes and CEAs can be improved by enrichment of EPSCs, which can form an ideal self-renewing epidermis and maintain the capacity to respond to local signaling in a spatiotemporal fashion [114, 115]. In addition, a culture of EPSCs on a bed of fibroblasts embedded with a plasma matrix has been used, which enables restoration of both the epidermal and dermal compartment [116]. Furthermore, two recent animal studies have pointed to a possible alternative to the method used traditionally. Activation of EPSCs from the HF bulge in mice with third-degree burns induced with human α-defensin-5 derived from the intestine accelerated wound healing and importantly induced hair regeneration [117]. Similarly, transplantation of LGR6-positive EPSCs isolated by fluorescence-activated cell sorting and administered by injection into the wound significantly promoted re-epithelization, hair growth, and angiogenesis [118]. The clinical performance of EPSCs may be improved by the use of carrier substrates that mimic the mechanical properties of the niches, providing a normal physiological environment that can optimize the regenerative function of EPSCs.

#### Chronic wounds

The number of patients with chronic wounds, even non-healing wounds, has increased with the increased prevalence of diabetes, obesity, and vascular disease and an aging population. The commonly used therapeutic strategies are similar to those for burns, such as skin substitutes and CEAs. However, due to the complicated pathogenesis of chronic wounds, many skin substitutes and grafts have been demonstrated to be useless [119]. Excessive inflammation and dysregulation of matrix metalloproteinases prevent normal ECM remodeling and wound healing. In addition, chronic wounds also lead to compromised local EPSC populations that become depleted through frequent cycling and an inability to regenerate the epidermis because of a hostile microenvironment [120]. However, this negative cycle may be broken when chronic wounds are transplanted with CEAs enriched with EPSCs on an ECM-compatible substrate, which can simultaneously overcome the deficiency of EPSCs and provide ECM components to stabilize the wound site [5]. In addition, a functional epidermis has also been regenerated on previously infected non-healing skin ulceration by a discrete number of gene-corrected EPCs [121].

#### Other epithelial regeneration

The production of replacement tissue or organs and biologically compatible constructs is highly warranted because of the shortage of donor organs. Autologous EPSCs exhibit plasticity, which can differentiate into all three embryonic germ layers when injected into mouse blastocysts [122]. In addition, EPSCs also express cornea-specific cytokeratin when cocultured with corneal cells or eye-specific stromal ECM [123]. Therefore, EPSCs are considered an ideal source for the replacement of damaged epithelia.

### Urethral regeneration

Skin grafts have become an alternative method for urethral regeneration because they are adaptable in an environment of urine exposure. However, the hair will grow in the urethral lumen in later years after transplantation [123]. Cultured urethral epithelium from the bladder is another approach, but it is an invasive procedure and causes additional injury. Thus, employing CEAs enriched with EPSCs has been considered an ideal approach for the restoration of a functional urethra, which not only provides epithelial cells that do not grow hair but can also be harvested easily without secondary damage [124].

### Limbal stem cell deficiency (LSCD)

LSCD is caused by damage or loss of limbal SCs and often leads to blindness. There are many similarities between corneal and skin epithelia, such as a typical stratified epithelial morphology and expression of SC marker p63 [125]. An animal study has indicated that skin EPSCs can partially or even fully restore a clear cornea, and the reconstructed corneal epithelium expresses the eye-specific markers KRT3, KRT12, and paired box (PAX)6 but does not express skin-specific KRT10 [123]. In addition, CEAs with prior genetic modification, such as PAX6, can re-establish a clear cornea, even after repeated corneal scraping [126]. The above research suggests that the plasticity and regenerative capacity of EPSCs enable regeneration of epithelium in other organs.

### Stable vitiligo

Vitiligo is a common skin disease characterized by the loss of melanocytes. Split-skin grafting is a common therapeutic approach that often results in a pitted skin surface and does not always improve pigmentation. CEA combined with or without melanocytes has been used in clinical trials. When CEA and a physiologically relevant number of melanocytes are transplanted, they integrate well with existing skin, color matching is good, and wound healing occurs without scarring [127]. In addition, repigmentation of achromatic lesions is achieved in 50–90% of cases after the application of CEA without melanocytes followed by exposure to sunlight [128].

### Gene therapy of epidermolysis bullosa

Epidermolysis bullosa is a severe skin disease caused by genetic mutations, such as laminin 5 or collagen 7 deficit [129]. Gene therapy consists of extracting EPSCs from patients with genetic abnormalities, correcting the mutation in vitro by gene transfer, and transplanting the cells back into the patient’s skin [130]. Clinical studies have shown that functional laminin-5 can be detected in patients, together with a normal adherent epidermis in the transplanted areas [131], suggesting that gene therapy using EPSCs is a promising therapeutic strategy in genetic diseases.

### Strategies to improve biological potential of EPSCs

The traditional mode of cell delivery to wounds, including topical EPSC applications, direct injections, and systemic delivery into circulation, has many limitations, such as low survival, high attrition rate, additional tissue damage, and lack of cell-ECM attachment [132]. Thus, the bioscaffold-based stem cell delivery strategy has gained considerable attention; in this strategy, cells are seeded on a matrix (such as hydrogel, scaffold, dermal substitute) first, and the matrix containing stem cells is then applied to the wound [107]. Polymer-based scaffolds show high bioactivity, biocompatibility, and biodegradability [133], which can be fabricated using natural polymers (e.g., hyaluronan, chitosan and alginate, collagen, elastin, fibrin, and silk [134]) and synthetic polymers (e.g., poly(lactic-co-glycolic) acid, polyanhydrides, polyethylene glycol), or genetically engineered peptides [135, 136]. Recently, modifications of classical scaffolds, such as addition of natural proteins (laminin, fibrin, glycosaminoglycan) or silver nanoparticles to the cellular dressing, have been shown to further improve wound healing by promoting the biological activity of stem cells [137, 138]. Recently, cultured epidermal cell sheets (CES) have been used to treat patients with skin injuries [139], and studies suggest that enrichment for EPSCs within CES may further improve wound healing, prevent hypertrophic scar formation, and provide long-term regeneration [140, 141]. In addition, 3D bioprinting is an emerging technology that can generate customized composite skin products [142]. By mimicking skin structures, this method provides microenvironmental niche architecture for the maintenance and growth of stem cells [143]. Thus, 3D-printed skin is an ideal scaffold for EPSCs.

Since EPSCs have the potential to regenerate skin, the genetic modification of EPSCs represents a novel treatment option. Studies have reported that transduction of EPCs with the laminin subunit β3 (LAMB3) gene from a patient suffering from junctional epidermolysis bullosa led to the successful completion of epidermal regeneration [121]. Our research group also found that EPSCs modified with epidermal growth factor (EGF) or Caveolin-1 showed increased ability to promote re-epithelialization, fibroblast proliferation, and wound healing [144]. In addition, pretreatment of EPSCs with curcumin not only promoted the proliferative ability of EPSCs but also enhanced the ability of conditioned medium from curcumin-treated EPSCs to accelerate wound closure [145].

Another trend in regenerative medicine is to use stem cell secretomes instead of cells themselves, which resolves some technical problems, such as tumorigenicity, cell immunogenicity, and infection transmission. For example, MSC-derived exosomes positively affect wound healing by promoting angiogenesis, cell migration, proliferation, and re-epithelialization process [146]. As products of pluripotent stem cells, EPSC-derived exosomes are highly promising as a novel therapy against skin injuries.

## Conclusions and perspectives

Due to the characteristics of EPSCs, such as large numbers, accessibility, and multipotency in the formation and differentiation of the epidermis, the use of these cells exhibits promise as an effective tissue repair strategy. Recent data have demonstrated the feasibility of autologous EPSC therapy in cutaneous repair and regeneration. Although there are still many unresolved questions regarding the experimental and clinical application of EPSCs, such as complex techniques and high cost, it is likely that in the future, knowledge of the biology of EPSCs and safety of the techniques will increase, allowing a more widespread application of EPSCs in wound healing and tissue regeneration.

## Data Availability

Data sharing is not applicable to this article as no datasets were generated or analyzed during the current study.

## Abbreviations

CEAs: Cultured epidermal autografts
CM: Conditioned (culture) medium
DNMT: DNA methyltransferase
ECM: Extracellular matrix
EMT: Epithelial-to-mesenchymal transition
EPSCs: Epidermal stem cells
EVs: Extracellular vesicles
Ezh: Enhancer of zeste
FGFs: Fibroblast growth factors
H3K27: H3 lysine 27
HDACs: Histone deacetylases
HMTs: Histone methyltransferases
IFE: Interfollicular epidermis
IL: Interleukin
KRT: Keratin
LGR: Leucine-rich-repeat-containing G protein-coupled receptor
LRIG: Immunoglobulin-like domains
LSCD: Limbal stem cell deficiency
PAX: Paired box
PDGF: Platelet-derived growth factor
PRDM: PR/SET domain
SCs: Stem cells
SGs: Sebaceous glands
SOX: SRY-box
TCF: Transcription factor
TNF: Tumor necrosis factor
UHRF: Depletion of ubiquitin-like with PHD and ring finger
