# Correction to: Epidermal stem cells in wound healing and their clinical applications

**DOI:** 10.1186/s13287-020-01960-9

**Published:** 2020-10-22

**Authors:** Ronghua Yang, Fengxia Liu, Jingru Wang, Xiaodong Chen, Julin Xie, Kun Xiong

**Affiliations:** 1 Department of Burn Surgery, The First People’s Hospital of Foshan, Foshan, 528000 China; 2 Department of Human Anatomy, School of Basic Medical Science, Xinjiang Medical University, Urumqi, 830001 China; 3 Department of Burn Surgery, First Affiliated Hospital of Sun Yat-Sen University, Guangzhou, 512100 China; 4 Department of Anatomy and Neurobiology, School of Basic Medical Science, Morphological Sciences Building, Central South University, 172 Tongzi Po Road, Changsha, 410013 Hunan China

**Correction to: Stem Cell Res Ther (2019) 10:229**

**https://doi.org/10.1186/s13287-019-1312-z**

The original article [1] omits an acknowledgement of permission to reproduce the original version of Figure 1 (published in *Actas Dermosifiliogr*, 2015 [2]) in this article.

**Fig. 1:**
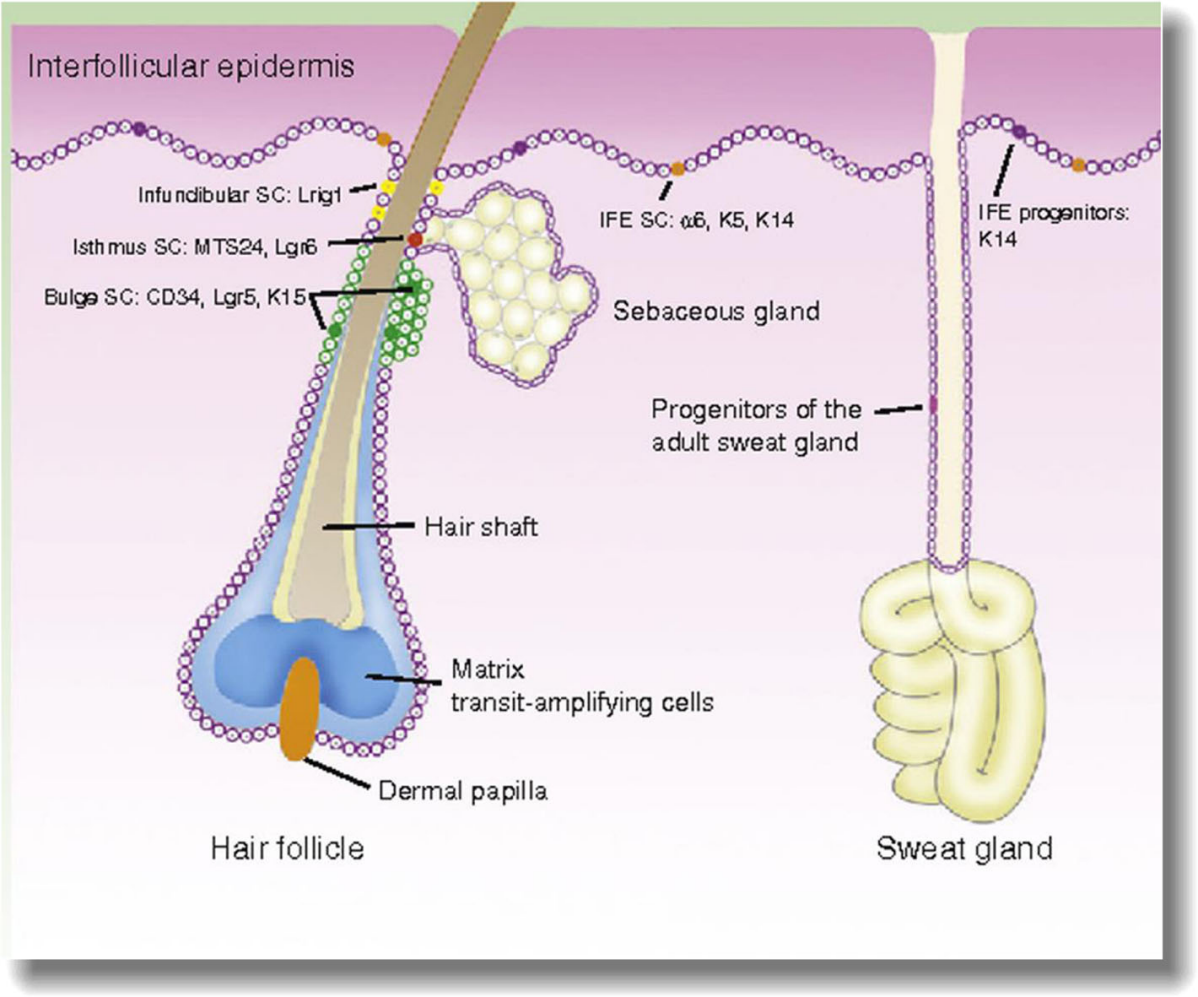
Illustration of the different populations of EPCs and their specific markers. Figure 1 was published with permission of the Publisher. Original source: Pastushenko I, Prieto-Torres L, Gilaberte Y, Blanpain C. Skin Stem Cells: At the Frontier Between the Laboratory and Clinical Practice. Part 1: Epidermal Stem Cells. Actas Dermosifiliogr. 2015;106:725-32. © 2015 Elsevier España, S.L.U. and AEDV. All rights reserved

The authors wish to note the following in this correction article:

Figure 1 was published with permission of the Publisher. Original source: Pastushenko I, Prieto-Torres L, Gilaberte Y, Blanpain C. Skin Stem Cells: At the Frontier Between the Laboratory and Clinical Practice. Part 1: Epidermal Stem Cells. Actas Dermosifiliogr. 2015;106:725-32. © 2015 Elsevier España, S.L.U. and AEDV. All rights reserved.
